# A body beaten again: a narrative analysis of a series of cases of breast cancer survivors punctuated by violence

**DOI:** 10.1192/j.eurpsy.2024.438

**Published:** 2024-08-27

**Authors:** V. S. D. Melo, C. Soares

**Affiliations:** ^1^Psychiatry, Centro Hospitalar do Médio Tejo, Tomar; ^2^Psychiatry, Instituto Português de Oncologia - Porto, Porto, Portugal

## Abstract

**Introduction:**

Various mechanisms have been identified to explain the relationship between gender-based violence, screening, and cancer. Biological mechanisms, primarily related to chronic stress and allostatic load, have been associated with high rates of chronic diseases among victims of violence, impairing the functioning of the immune and endocrine systems. Victims of abuse simultaneously show less initiative for screening exams, such as mammograms, as they perceive them as invasive and retraumatizing. They also demonstrate a greater tendency toward maladaptive coping behaviors and unhealthy lifestyles, such as abusive substance use. A significant number of these patients develop psychosocial dysfunction and body image disturbance during breast cancer treatments.

**Objectives:**

This work aims to provide a descriptive and narrative analysis of body image and psychosocial changes in women breast cancer survivors with prolonged experiences of violence, supported by a non-systematic literature review on the central aspects under study.

**Methods:**

For the introductory literature review, a search was conducted on search engines such as Google Scholar and PubMed, with no date limitations, using the following terms (or combinations): “intimate partner violence,” “violence AND cancer,” “body image AND psychossexual adjustment AND breast cancer.” Additionally, a narrative analysis of body image and psychosocial changes in women breast cancer survivors with prolonged experiences of violence was conducted. For this purpose, participants were asked to complete two validated scales in the Portuguese language, and first-person testimonials were collected.

**Results:**

The analysis of scale results and participant testimonials highlights a consensus on the significant impairment of psychosocial functioning and the experience of sexuality. There is evidence of avoidance behaviors in terms of affectionate and sexual contact due to feelings of fear, shame, and discomfort. The breast is valued as a sensual, erotic, and essential sexual element, and impactful changes in body image persist. However, in some cases, these changes are experienced as transformative and liberating, fostering a more generous view of the body, identity, and femininity.

**Conclusions:**

Women with breast cancer should be screened for the possibility of being victims of violence, as this context predicts a higher likelihood of emotional difficulties during surgical treatments, including psychological distress, post-traumatic stress, body shame, and self-blame. A significant number of women, including those in this study, consider the approach to self-image and sexuality in oncology consultations deficient. Psychological programs and interventions should be developed to empower patients to adjust to the sexual changes arising from treatments and disease progression and to promote positive intimate relationships and effective communication.

**Disclosure of Interest:**

None Declared

